# Multi-omic data integration for the study of production, carcass, and meat quality traits in Nellore cattle

**DOI:** 10.3389/fgene.2022.948240

**Published:** 2022-10-21

**Authors:** Francisco José de Novais, Haipeng Yu, Aline Silva Mello Cesar, Mehdi Momen, Mirele Daiana Poleti, Bruna Petry, Gerson Barreto Mourão, Luciana Correia de Almeida Regitano, Gota Morota, Luiz Lehmann Coutinho

**Affiliations:** ^1^ Department of Animal Science, Luiz de Queiroz College of Agriculture, University of São Paulo, Piracicaba, Brazil; ^2^ Department of Animal and Poultry Sciences, Virginia Polytechnic Institute and State University, Blacksburg, VA, United States; ^3^ Department of Agri-Food Industry, Food and Nutrition, University of São Paulo, Piracicaba, Brazil; ^4^ Department of Veterinary Medicine, School of Animal Science and Food Engineering, University of Sao Paulo, Pirassununga, Brazil; ^5^ Embrapa Pecuária Sudeste, São Carlos, Brazil

**Keywords:** Bayesian network, factor analysis, meat quality, latent variables, omics data

## Abstract

Data integration using hierarchical analysis based on the central dogma or common pathway enrichment analysis may not reveal non-obvious relationships among omic data. Here, we applied factor analysis (FA) and Bayesian network (BN) modeling to integrate different omic data and complex traits by latent variables (production, carcass, and meat quality traits). A total of 14 latent variables were identified: five for phenotype, three for miRNA, four for protein, and two for mRNA data. Pearson correlation coefficients showed negative correlations between latent variables miRNA 1 (mirna1) and miRNA 2 (mirna2) (−0.47), ribeye area (REA) and protein 4 (prot4) (−0.33), REA and protein 2 (prot2) (−0.3), carcass and prot4 (−0.31), carcass and prot2 (−0.28), and backfat thickness (BFT) and miRNA 3 (mirna3) (−0.25). Positive correlations were observed among the four protein factors (0.45–0.83): between meat quality and fat content (0.71), fat content and carcass (0.74), fat content and REA (0.76), and REA and carcass (0.99). BN presented arcs from the carcass, meat quality, prot2, and prot4 latent variables to REA; from meat quality, REA, mirna2, and gene expression mRNA1 to fat content; from protein 1 (prot1) and mirna2 to protein 5 (prot5); and from prot5 and carcass to prot2. The relations of protein latent variables suggest new hypotheses about the impact of these proteins on REA. The network also showed relationships among miRNAs and nebulin proteins. REA seems to be the central node in the network, influencing carcass, prot2, prot4, mRNA1, and meat quality, suggesting that REA is a good indicator of meat quality. The connection among miRNA latent variables, BFT, and fat content relates to the influence of miRNAs on lipid metabolism. The relationship between mirna1 and prot5 composed of isoforms of nebulin needs further investigation. The FA identified latent variables, decreasing the dimensionality and complexity of the data. The BN was capable of generating interrelationships among latent variables from different types of data, allowing the integration of omics and complex traits and identifying conditional independencies. Our framework based on FA and BN is capable of generating new hypotheses for molecular research, by integrating different types of data and exploring non-obvious relationships.

## Introduction

Meat quality traits, which include meat tenderness, are an important aspect for consumers and are related to the customer’s acceptability and beef repurchase (Rust et al., 2008). Meat quality traits are complex and influenced by diet, pre- and post-slaughter management, meat processing, storage methods, genetic factors, and genotype-by-environment interaction (Wheeler et al., 2005; Adzitey, 2011; Guerrero et al., 2013; Njisane and Muchenje, 2016). Most of the biological mechanisms involved in meat quality traits are not completely understood. In this context, systems biology has been proposed to elucidate the flux of molecular information, generating a holistic point of view for complex traits (Ideker et al., 2001). Data from the genome, transcriptome, proteome, microRNAome, and metabolome have been used independently to study the molecular architecture of complex traits and identify important genes, pathways, and networks that underlie economic livestock traits in the last decade (Tizioto et al., 2013; Carvalho et al., 2014; Cesar et al., 2014, 2016; Novais et al., 2019). However, studies using single omic data disregard the interactions among different levels of biomolecules, postulated by the central dogma of molecular biology (Ritchie M. D. et al., 2015).

Complex traits are regulated at different molecular levels, and considerable effort has been made to generate multi-level studies, integrating different omic data to understand the inherent biological meaning of livestock traits (Widmann et al., 2013; Suravajhala et al., 2016). However, omic data integration using a hierarchical analysis approach or considering just the common pathway enrichment may not reveal non-obvious relationships that exist among omic data (Misra et al., 2018). In this context, efforts to develop approaches to data omic integration have been proposed (Huang et al., 2017).

Factor analysis (FA) reduces the dimensionality of data, inferring latent (hidden) variables to explain dependencies among observed variables that share common variations (Meng et al., 2016). Furthermore, the Bayesian network (BN) has the potential to generate relationships among phenotypes and molecules by a graph-based model of joint multivariate probability distributions that represent conditional independence between variables (Rodin and Boerwinkle, 2005). Here, phenotypes of production, carcass, meat quality, and multi-omic data were fitted into the FA and BN framework to explore the potential biological interrelationships to generate new hypotheses for complex traits in beef cattle.

## Materials and methods

### Animals and phenotypes

A total of 386 Nellore steers born between 2009 and 2011 at the Brazilian Agricultural Research Corporation (EMBRAPA/Brazil) were initially included in this study. The animals were raised in feedlots under identical diets, and environmental conditions, and slaughtered at age of 25 months. More details regarding animals, diet, and experimental design can be found in Cesar et al. (2014). The animals were handled and managed according to the Institutional Animal Care and Use Committee Guidelines from the Brazilian Agricultural Research Corporation—EMBRAPA approved by the president, Dr. Rui Machado.

Carcass ultrasound evaluations were performed by trained field technicians and followed the standards set by the Ultrasound Guidelines Council (UGC; www.ultrasoundbeef.com). An Aquila Pie Medical (Pie Medical Inc., Maastricht, Netherlands) equipped with a 172 mm-long linear transducer with a frequency of 3.5 MHz was used to measure the initial ribeye area (REAi) and initial backfat thickness (BFTi) obtaining sectional images of the longissimus dorsi (LD) muscle between the 12th and 13th ribs. The images were stored and measurements were obtained by ODT Eview R (Pie Medical Inc., Maastricht, Netherlands).

The details of carcass and meat quality trait evaluations were previously described by Nascimento et al. (2016). The visceral organs were removed during slaughter, and the heart, kidney, liver, and perirenal, pelvic, and inguinal fats were weighed. Carcasses were weighed and chilled for 24 h at 5°C. The carcass was weighted at 24 h, and the carcass depth was measured on the fifth rib from top to bottom, measuring the distance from the sternum to the middle of the spine where the marrowbone passes.

Steaks of 2.54 cm thick from the LD muscle between the 12th and 13th ribs were collected 24 h after slaughter. Steaks were vacuum packed and used to measure the shear force (SF; Kg), backfat thickness (BFT; mm), ribeye area (REA; cm^2^), myofibrillar fragmentation index (MFI), color parameters (L* = lightness, a* = redness, and b* = yellowness), intramuscular fat (IMF; percentage), pH at 24 h, moisture, water holding capacity, and cook loss. Briefly, the final backfat thickness (BFTf) was measured using a ruler in millimeters (Filho, 2000). Color parameters L*, a*, and b* were measured after exposing the steaks to atmospheric oxygen for 30 min prior to analysis using a Hunter Lab colorimeter model MiniScan XE with Universal Software v. 4.10 (Hunter Associates Laboratory, Reston, VA), illuminant D65, and 10° standard observer. Additionally, muscle pH was measured at three locations across the steak using a Testo pH measuring instrument model 230 (Testo, Lenzkirch, Germany). The final ribeye area (REAf) was calculated as the area of LD muscle using a grid. Cooking losses were measured as the weight difference between the steaks before and after cooking. For IMF, approximately 100 g of muscle samples, previously lyophilized and ground, were obtained using an Ankom XT20 extractor as described in AOCS official procedure Am 5-04 (Horwitz, 2000). The myofibrillar fragmentation index was determined according to Hopkins et al. (2000). The SF values were obtained from 2.54 cm thick steaks after 24 h of aging at 2°C in a cold chamber using the texture analyzer TA-XT2i coupled to a Warner–Bratzler blade with 1.016 mm thickness.

### mRNA data processing and WGCNA

For total RNA extraction, a sample of 100 mg of the LD muscle was processed using the Trizol reagent (Life Technologies, Carlsbad, CA, United States), following the manufacturer’s guidelines. After extraction, RNA integrity was verified using the Bioanalyzer 2100 (Agilent, Santa Clara, CA, United States), and the samples presenting RNA integrity numbers lower than 7.0 were removed from further analysis. A total of 2 µg of RNA from each sample was used for the cDNA library preparation, in accordance with the protocol described in the TruSeq RNA Sample Preparation kit v2 guide (Illumina, San Diego, CA, United States). The libraries were sequenced using the HiSeq2500 ultra-high-throughput sequencing system (Illumina, San Diego, CA, United States) with the TruSeq SBS kit v3-HS (200 cycles). All sequencing analyses were performed at the ESALQ Genomics Center (Piracicaba, São Paulo, Brazil).

The FastQC software v0.10.1 (https://www.bioinformatics.babraham.ac.uk/projects/fastqc/) was applied to check the quality of the sequencing data. Low-quality reads were filtered and adapter sequences were trimmed using Seqyclean package version 1.4.13 (Neapolitan, 2004). The details of data acquisition were previously described by de Lima et al. (2020).

The read alignment was carried out against the bovine reference genome *Bos taurus* ARS-UCD1.2 (available at the Ensembl database https://www.ncbi.nlm.nih.gov/assembly/GCF_002263795.1) and read counts using STAR software (Spliced Transcripts Alignment to a Reference) version 2.7 (Dobin and Gingeras, 2015) with the Ensembl (release 95, January 2019) gene annotation file. Subsequently, genes with zero counts for all samples were removed. Next, the genes were filtered by the counts different from zero in at least 70% of the samples and counts per million (CPM) > 5 using the EdgeR Bioconductor package (Chen H.-J. et al., 2018). This was followed by normalizing counts using the DESEq2 Bioconductor package (Love et al., 2014), and a batch effect was identified using the limma R package (Ritchie M. E. et al., 2015).

Clustering analysis was performed on the mRNA dataset using the weighted gene co-expression network analysis (WGCNA) R package (Langfelder and Horvath, 2008). To measure the connectivity among genes, an adjacency matrix was generated by calculating the Pearson’s correlation coefficients among all genes and raising it to a power *ß* (soft threshold) of 6, which is chosen using a scale-free topology criterion (*R* ^2^ = 0.8). Modules containing at least 30 genes were retained. Modules with hub genes that had a module membership (MM) > 0.95 and gene significance (GS) with a *p*-value < 0.001 were kept for further analysis. Enrichment analysis was performed using MetaCore software (MetaCore, 2021) to elucidate biological processes and pathways represented by the hub genes of modules.

### miRNA and data acquisition

Small RNA libraries were constructed from 1 μg of total RNA from each sample using the Illumina TruSeq small RNA Sample Prep Kit (Illumina Inc, San Diego, CA, United States), in accordance with the manufacturer’s protocol. High Sensitivity DNA Chip and an Agilent 2100 Bioanalyzer (Agilent Technologies) was used to determine library quality and qPCR with the KAPA Library Quantification kit (KAPA Biosystems, Foster City, CA, United States) for quantification. Sequencing was performed using a Miseq Reagent Kit v3 for 150 cycles in an Illumina Miseq Sequencing System (Illumina Inc., San Diego, CA, United States). The Illumina CASAVA v1.8 was used to generate and de-multiplex the raw fastq sequences. The quality of Illumina deep sequencing data was determined using the FastQC program (version 0.9.5) (Andrews, 2010). Adapters and low-quality reads were trimmed using Cutadapt (version 1.2.1) (Martin, 2011). Filtered reads were then processed following the mirDeep2 analysis pipeline (Friedländer et al., 2012). Sequences were aligned to the *Bos taurus* ARS-UCD.1.2 reference genome (available at the Ensembl database (https://www.ncbi.nlm.nih.gov/assembly/GCF_002263795.1). Only alignments with zero mismatches in the seed region (first 18 nucleotides of a read sequence) of a read mapped to the genome were retained. More details about data acquisition were provided by Kappeler et al. (2019).

Briefly, miRNAs with zero counts for all samples were removed. Next, the miRNAs were filtered by the counts that are different from zero in at least 70% of the samples and CPM >5 using the EdgeR Bioconductor package (Chen Y. et al., 2018). The miRNA counts were normalized using the DESEq2 Bioconductor package (Love et al., 2014), and the limma R package was used to identify a batch effect (Ritchie M. E. et al., 2015).

### Proteome and data acquisition

The details for data acquisition and processing are previously described in Poleti et al. (2018). Frozen muscles (500 μg) of 106 animals were ground on liquid nitrogen, then transferred to a microcentrifuge tube, and weighed to minimize protein degradation. The muscle was homogenized in 2.5 ml lysis buffer containing 8 M urea , 2 M thiourea, 1% DTT, 2% CHAPS, and 1% protease inhibitor cocktails (Sigma-Aldrich) in an ULTRA-TURRAX^®^ IKA homogenizer on ice for 2 min. The extracts were vigorously shaken for 30 min on ice and centrifuged at 10,000 x g for 30 min at 4°C. The supernatants were collected, the total protein concentration was determined by the PlusOne 2-D Quant Kit (GE Healthcare), and then stored at −80°C for further analysis.

The protein extract was desalted with a 3-kDa cutoff Amicon^®^ Ultra centrifugal filter (Millipore, Ireland), where the lysis buffer was exchanged using a solution of 50 mm ammonium bicarbonate and 2 M urea five times. The concentration of the retained protein solution was quantified using a Bradford Protein Assay Kit (BioRad). For protein digestion, 50 μg of proteins of each sample were denatured with 25 μL of 0.2% RapiGest SF (Waters Corporation, United States) at 80°C for 15 min, reduced with 2.5 μL of 100 mm dithiothreitol (DTT) (Sigma, United States) at 60°C for 30 min, and alkylated with 2.5 μL of 300 mm iodoacetamide (AA) (Sigma, United States) at room temperature in the dark for 30 min. Enzymatic digestion was performed with sequencing grade modified trypsin (Promega) at a 1:100 (w/w) enzyme: protein ratio at 37°C for 16 h. Digestion was stopped by the addition of 10 μL of 5% (V/V) trifluoroacetic acid and incubated at 37°C for 90 min to hydrolyze the RapiGest (Yu et al., 2003). The peptide mixture solution was then centrifuged at 18,000 x g for 30 min at 6°C. The supernatant was transferred to a new vial, dried down in a vacuum centrifuge, and stored at −20°C.

Qualitative and quantitative bidimensional nanoUPLC tandem nanoESI-HDMSE analyses were conducted using both 1-h reversed-phase gradient from 7% to 40% (v/v) acetonitrile (0.1% v/v formic acid) and 500 nL*min^−1^ on a nanoACQUITY UPLC 2D Technology system (Gilar et al., 2005). A nanoACQUITY UPLC HSS T3 1.8 μm, 75 μm × 15 cm column (pH 3) was used in conjunction with a reverse-phase (RP) XBridge BEH130 C18 5 μm 300 μm × 50 mm nanoflow column (pH 10). The ion mobility cell was activated and filled with nitrogen gas, which operates at the cross-section resolving power of at least 40 Ω/ΔΩ (Lalli et al., 2013). The effective resolution has the conjoined ion mobility of >1.5 M FWHM (Silva et al., 2014). The ionization of samples was performed using a NanoLockSpray ionization source (Waters, Manchester, United Kingdom) in the positive ion mode nanoESI (+). The mass spectrometer was calibrated with an MS/MS spectrum of [Glu1]-fibrinopeptide B human (Glu-Fib) solution (100 fmol*uL^−1^) delivered through the reference sprayer of the NanoLockSpray source. Data acquisition was performed using a Synapt G2-S HDMS mass spectrometer (Waters, Manchester, United Kingdom). A mass–charge value ranges from m/z 50 to 2000.

Mass spectrometry data were acquired with Waters MassLynx v.4.1 software and processed using Progenesis QI for Proteomics (QIP) 2.0 software (Nonlinear Dynamics, United Kingdom). Progenesis QIP software was used to run alignment, peak picking, ion drift time data collection, ion abundance measurements, normalization, quantification, peptide and protein identification, and statistical analysis. The processing parameters for Progenesis included the following: automatic tolerance for precursor and product ions based on peptide identification and normal distribution (Geromanos et al., 2009), one missed cleavage, carbamidomethylation of cysteine as a fixed modification, and oxidation of methionine as variable modification. For protein identification and quantification, the obtained raw data were searched against a Nellore transcriptome database built from RNA-sequencing data from LD muscle. Data quality assessment was performed accordingly (Souza et al., 2017), and proteins were selected based on the detection and identification in at least 80% of biological samples. The assembled data were compared to the NCBI’s UniProt database (https://www.uniprot.org/) as functional analysis.

### Factor analysis

This section closely follows the work of Yu et al. (2020) and Momen, et al. (2021). The exploratory factor analysis (EFA) was applied to search the structure of underlying latent variables (factors) that drive the observed phenotypes and omic data. First, the caret R package (Kuhn, 2008) was used to check collinearity, and one of the features with correlation >0.9 was removed. Then, the Kaiser–Meyer–Olkin (KMO) test was applied to measure the sampling adequacy using the psych R package (Revelle, 2017) assessing the factor ability of the data (Cerny and Kaiser, 1977). The measure of sampling adequacy ranges between 0 and 1, and values closer to 1 are preferred. Here, KMO >0.7 was considered acceptable. The number of underlying latent variables *q* was determined using a parallel analysis (Horn, 1965) using the psych R package, as described in more detail in a previous work of our group (Momen et al., 2021). The EFA model is given as a function of latent factor scores.

Y=ΛF+ε,

where Y is a *p × n* matrix of p molecular features or phenotypes of n animals, Λ is the *p × q* matrix of factor loading connecting the relation between features and latent common factors, F is the *q × n* matrix of latent factor scores, and ε is the *p × n* vector of unique effects that is not explained by q underlying common factors. The variance–covariance matrix of Y is

$$\Sigma =\Lambda \Phi \Lambda^{\prime }+\Psi ,$$

where Σ is the *p × p* variance–covariance matrix of phenotypes, Ф is the variance of factor scores, and Ѱ is a *p × p* diagonal matrix of unique variance. The elements of Λ, Ф, and Ѱ are parameters of the model to be estimated from the data. With the assumption of F ∼ Ɲ(0, I), Λ and Ѱ were estimated by maximizing the log-likelihood of ℒ (Λ, Ψ|Y) using the R package psych (Revelle, 2017) along with a varimax rotation (Kaiser, 1958). A parallel analysis was performed to determine the number of underlying factors. A feature having loading > |0.55| was assigned to only one of the factors based on the factor loadings.

The Bayesian confirmatory factor analysis (BCFA) is an alternative to frequentist CFA generating an important role in the assessment of the reliability and validity of latent variables. We fitted BCFA to estimate the factor scores according to the phenotype-factor structure inferred from the earlier EFA step. BCFA was applied to concatenated data, including phenotypes, proteins, miRNA, and the hub genes of modules obtained from WGCNA. Briefly, the blavaan R package (Merkle and Rosseel, 2018) was used with three Markov Monte Carlo chains, each with 6,000 Gibbs samples after 6,000 burn-in. Then, the posterior means of the factor scores of latent variables were estimated and treated as the new phenotypes for further analysis.

### Bayesian network

In the Bayesian network (BN), a direct acyclic graph is generated, and each random variable is associated with a node, the edges represent conditional dependency between variables, whereas the absence of an edge implies that the variables are conditionally independent of other variables (Choi, 2015). The details of BN procedures can be found in more detail in Yu et al. (2019) and Momen et al. (2021). Briefly, the BN structure learning with the bnlearn R package (Scutari, 2010) was applied to study the probabilistic relationships among the omic and latent variables. The BN is given by

$$BN=\left(G,X_{V}\right),$$

where G represents a direct acyclic graph composed of nodes (V) connected by edges (E), describing the probabilistic relationships and the vector $X_{V}$ = ( $X_{1},\;...\;,\;X_{k})$ where *k* is the random variable (Yu et al., 2019). The joint probability of distributions is therefore given by

$$P\left(X_{V}\right)=\overset{k}{\underset{v=1}{\prod }}P\left(X_{V}\;|P_{a}\left(X_{V}\right)\right),$$

where $P_{a}\left(X_{V}\right)$ expresses a set of parent nodes of *X*_*V*_. The score-based (hill climbing and tabu) and hybrid algorithms (max–min hill climbing and general 2-phase restricted maximization) were used to perform structure learning (Scutari, 2010). Candidate networks were compared based on the Bayesian information criterion (BIC) and Bayesian Gaussian equivalent score (BGe). The BIC score was calculated as a criterion for the selection of the candidate model, and BGe reflects the posterior probability of the networks. A larger BIC score is preferred since it is rescaled by −2 in the bnlearn R package. In addition, 1,000 bootstrapping replicates were used to estimate the uncertainty of the edge’s strength and the direction of the network. Edges showing presence in at least 80% (strength) among all the 1,000 models were kept in the BN through model averaging.

## Results

### Data preprocessing for analysis

In this study, we investigated the effective application of FA and BN framework to generate networks with biological meaning on for three different phenotypic categories: 1) production trait category included pre-feedlot body weight (BWi), post-feedlot body weight (BWf), initial backfat thickness (BFTi), and initial ribeye area (REAi); 2) carcass trait category included final backfat thickness (BFTf), final ribeye area (REAf), hot carcass weight (carcass_hot), cold carcass weight (carcass_cold), carcass depth (carcass_depth), kidney fat content (fat_kidney), and pelvis fat content (fat_pelvis); and 3) meat quality category included the shear force at 24 h (SF), pH at 24 h (pH), meat moisture (moisture), free water (water_free), water-holding capacity (w_ret_cap), cooking weight loss (cook_loss), color parameters (L*, a*, and b*), myofibrillar fragmentation index (MFI), and intramuscular fat (IMF) along with three different omic datasets: 1) mRNA sequencing, 2) miRNA sequencing, and 3) protein abundance.

**FIGURE 1:**
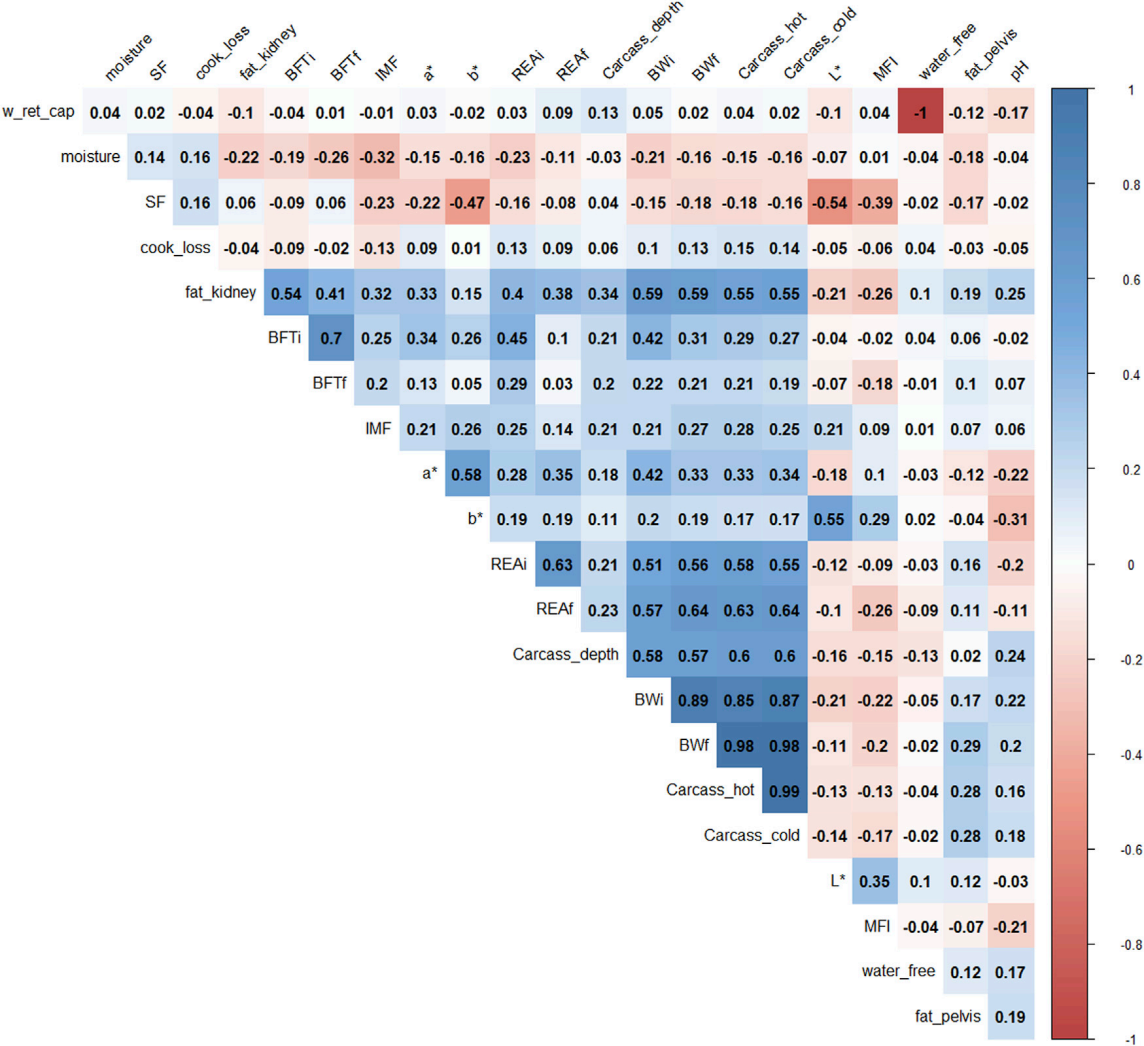
Correlation plot of 22 phenotypes. The degree of shading and the value reported correspond to the correlations among the traits. BWi: pre-feedlot body weight; REAi: initial ribeye area by ultrasonography; REAf: final ribeye area on steak; BFTi: initial backfat thickness by ultrasonography; BFTf: final backfat thickness by carcass; fat_pelvis: pelvis fat content at carcass; fat_kidney: kidney fat content; carcass_hot: hot carcass weight; carcass_cold: cold carcass weight; carcass_depth: carcass depth; pH: pH at 24 h; water_free: free water; w_ret_cap: water holding capacity; moisture: meat moisture; SF: shear-force; MFI: miofibrilar fragmentation index; L*, a*, b*: color parameters; and IMF: intramuscular fat.

Pearson’s correlations (Figure 1) showed that BWf, carcass_hot, and water_free were highly correlated with carcass cold and water-holding capacity (correlation >0.9), therefore; they were removed for further analysis to avoid duplicate information. For example, the correlation between water_free and w_ret_cap was −1 because both traits represent oppositional and complementary information (Pearce et al., 2011).

For the RNA-Seq (mRNA) data, after the quality control and filtering procedure, 13,023 genes were included in WGCNA. The WGCNA method identified 20 modules, and two modules (mRNA1 and mRNA2) showed module membership (MM) > 0.95 and gene significance *p*-value < 0.001. The mRNA1 module was composed of seven hub genes and the mRNA2 module of four hub genes (Supplementary Table S1).

**FIGURE 2:**
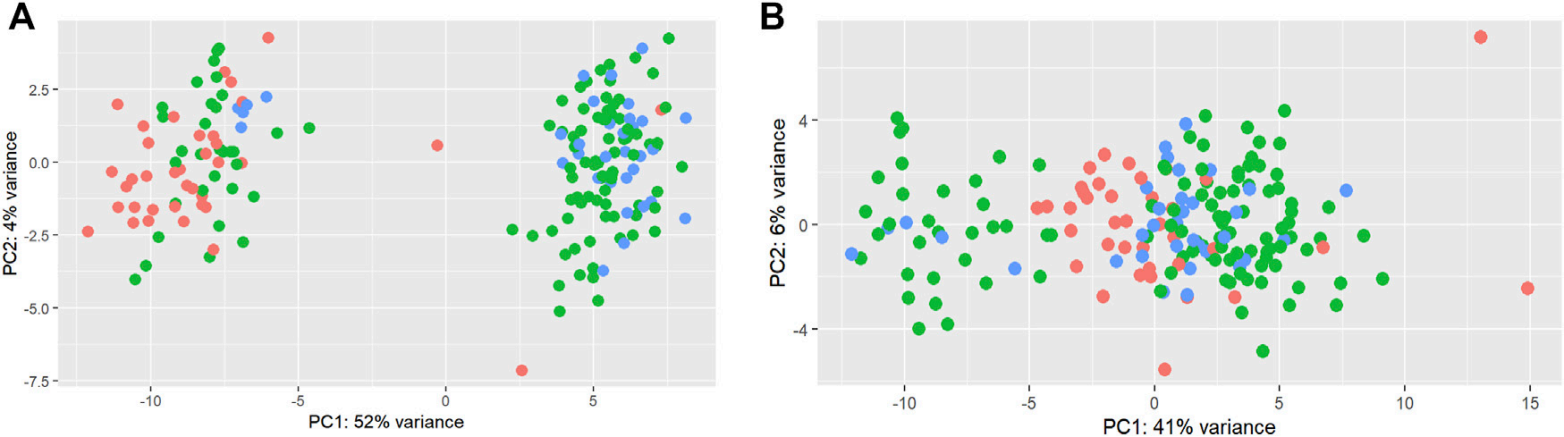
Principal component analysis of total counts as a batch effect in miRNAs. Principal component analysis of miRNAs before **(A)** and after **(B)** the limma batch effect normalization. The total counts refer to the total number of reads per sample. Three colors were used to represent 1) samples with a higher total number of reads: higher than mean +standard deviation (345,281 reads) (blue); 2) samples with a lower total number of reads: lower than mean—standard deviation (125,193 reads) (red); 3) samples with the average total number of reads: between mean > + standard deviation and mean < + standard deviation (green).

A total of 192 miRNAs were used for further analysis after the preprocessing steps. One animal was excluded as an outlier. After normalization, limma was used to identify a batch effect (Figure 2). Principal component analysis (PCA) revealed clusters based on the total counts of samples (Figure 2A). limma was applied to remove the batch effect in the miRNA data for further analysis (Figure 2B).

**FIGURE 3:**
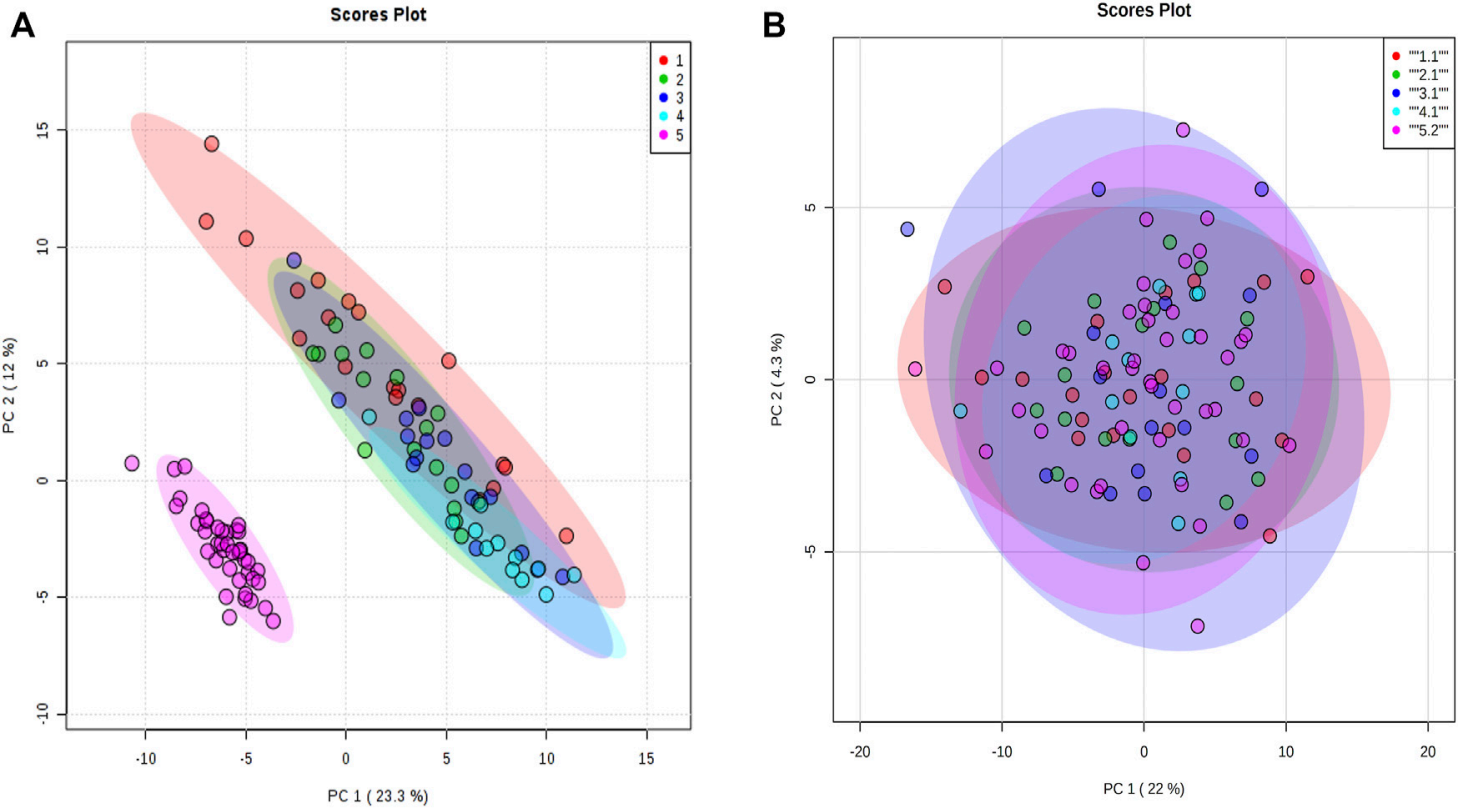
Principal component analysis of proteomic data. **(A)** Principal component analysis with all animals normalized together. **(B)** Principal component analysis when animals were normalized separately by equipment acquisition. The colors denote the equipment batch effect. The samples in red, green, dark blue, and blue colors are from equipment 1. The samples in pink are from equipment 2.

For proteomic data, 159 proteins from 106 animals were used in the analysis after the quality control steps. PCA was applied and a batch effect due to the equipment used was identified (Figure 3A). The batch effect was accounted for by normalizing every data separately (Figure 3B).

### Exploratory and Bayesian confirmatory factor analysis

**FIGURE 4:**
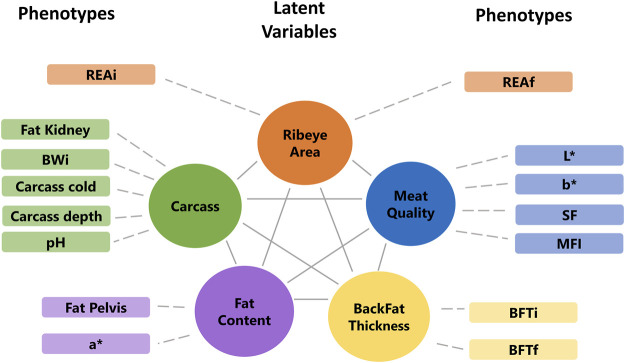
Final underlying latent structures of phenotypes generated by exploratory factor analysis. BWi: initial body weight on feedlot trial; REAi: initial ribeye area by ultrasonography; REAf: final ribeye area on steak; BFTi: initial backfat thickness by ultrasonography; BFTf: final backfat thickness by carcass; SF: shear-force; MFI: myofibrillar fragmentation index; and L*, a*, b*: color parameters.

**FIGURE 5:**
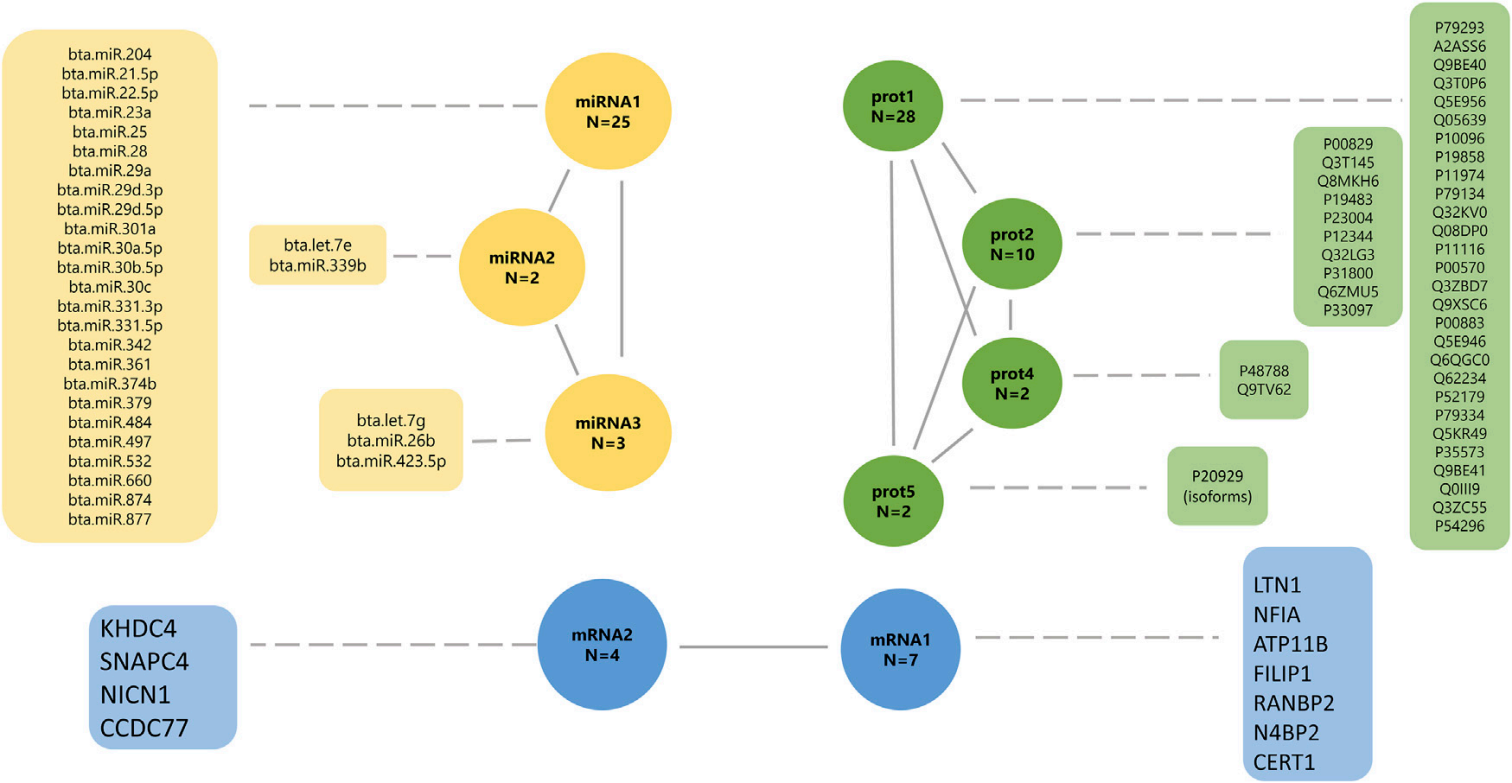
Final underlying latent structures of miRNA (yellow), proteins (green), and mRNA (blue) generated by exploratory factor analysis. N denotes the total number of features in each latent variable.

The factor analyses were performed using a subset of 102 animals that have phenotypes, miRNA, mRNA, and protein data. First, the phenotypes, miRNA, and protein data were used individually to fit an exploratory factor analysis (EFA). EFA can reduce data dimension without any prior assumptions about the observed data and latent factors structures. The parallel analysis suggested that phenotypes, miRNA, and protein data were composed of five, ten, and eight latent variables, respectively. Each omic dataset was assigned to a factor according to the highest loading value (>|0.5|), filtering some latent variables composed of a few features. The final underlying latent structures from EFA of the phenotype, miRNA, and protein data are shown in Figures 4, 5.

The BCFA was used to estimate factor loadings and scores based on the structure obtained from the EFA analysis, assuming that these latent variables determine the observed phenotypes and molecular profile levels (Supplementary Tables S2, S3).

The five phenotype latent factors showed strong contributions to the observed phenotypes, with standardized regression coefficients ranging from 0.989 to 0.986 for backfat thickness, −0.993 to 0.956 for meat quality, 0.654 to 1 for the carcass, 0.942 to 0.992 for fat content, and 0.973 to 0.991 for ribeye area. The seven latent variables for miRNA and protein also showed strong contributions to the molecular level profiles, with standardized regression coefficients ranging from -0.999 to 0.999 for factor mirna1 (miRNA), −0.971 to 0.979 for factor mirna2 (miRNA), −0.914 to 0.989 for factor mirna3 (miRNA), 0.842 to 0.990 for factor prot1 (protein), 0.774 to 0.973 for factor prot2 (protein), 0.963 to 0.997 for factor prot4 (protein), and 0.976 to 0.990 for factor prot5 (protein).

The latent factor backfat thickness (BFT) had a positive contribution to BFTi and BFTf (0.989 and 0.986, respectively; Supplementary Table S2), indicating that larger values for the latent factor can be interpreted as a greater thickness on the backfat content. The latent factor meat quality has a positive contribution to shear force (0.956; Supplementary Table S2), and a negative contribution to the colors b*, L*, and MFI (−0.993, −0.959, and −0.785, respectively) indicating that lower values on the latent factor can be interpreted as more tender meat. The latent factor carcass showed the largest positive contributions to traits describing carcass (e.g., weight to carcass cold, 1; weight to carcass depth, 0.990; weight to the kidney’s fat content, 0.987; and pH of meat at 24 h, 0.863), suggesting that this latent factor is an overall representation of carcass. The latent factor ribeye area (REA) has a strong positive contribution to the REAf and REAi (0.991 and 0.973, respectively; Supplementary Table S2), indicating that larger values for the latent factor can be interpreted as a greater ribeye area.

The latent factor mirna1 has a positive contribution to 18 miRNAs (0.876–0.999; Supplementary Table S2), and a negative contribution to seven miRNAs (−0.995 to −0.999, respectively). The mirna2 latent variable has a positive contribution to miRNA “bta.let.7e” (0.979; Supplementary Table S2), and a negative contribution to miRNA “bta.miR.339b” (−0.971, respectively; Supplementary Table S2). The latent factor mirna3 has a positive contribution of two miRNAs, “bta.let.7 g” (0.889) and “bta.miR.26b” (0.987), and a negative contribution to miRNA “bta.miR.423.5p” (−0.914). The latent factors prot1, prot2, prot4, and prot5 have a positive contribution to all proteins, including 28 proteins (0.842–0.990), 10 proteins (0.774–0.973), two proteins (0.976–0.997), and two proteins (0.976–0.990), respectively.

### Correlation among latent variables

**FIGURE 6:**
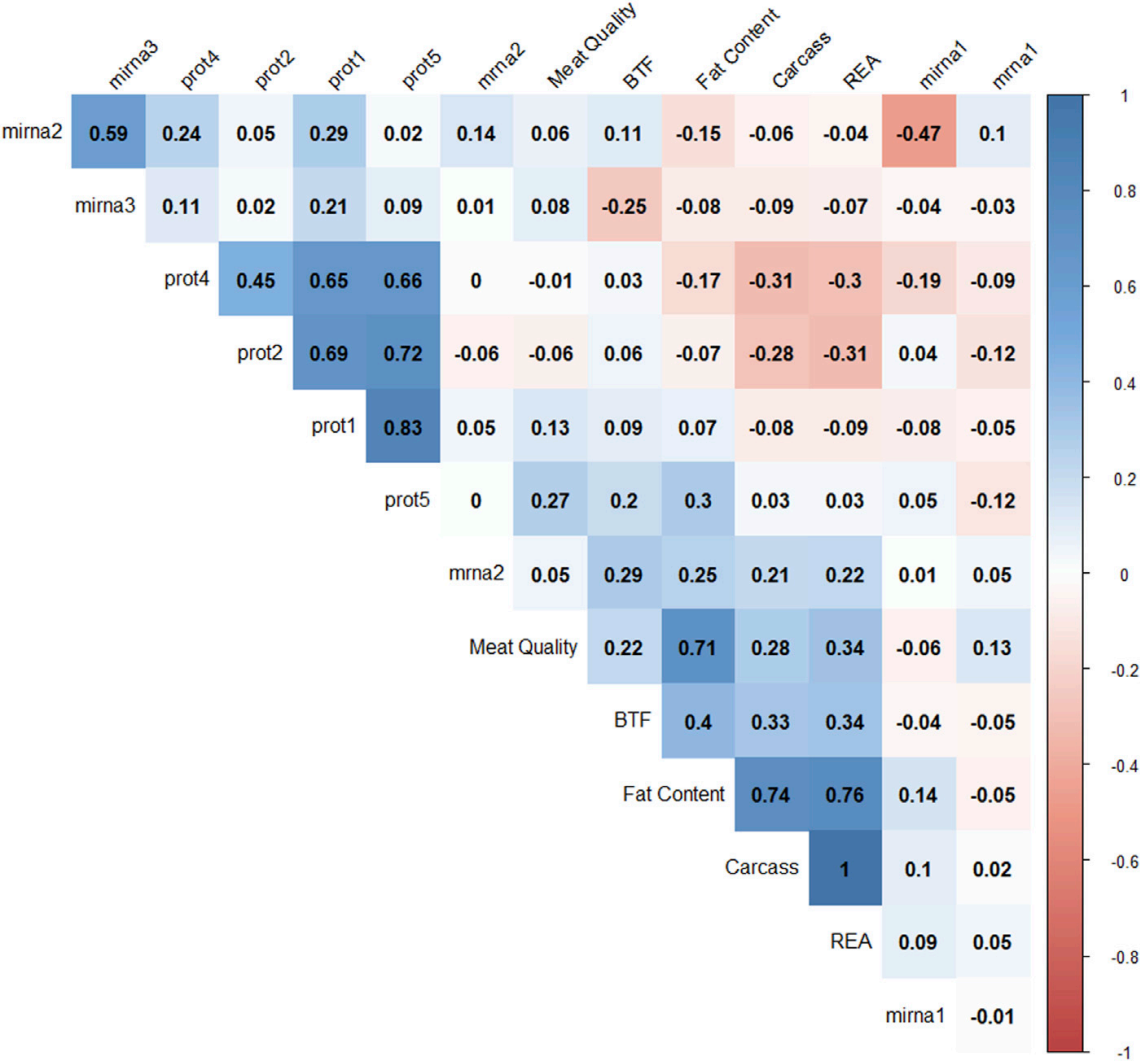
Correlation plot of 14 factor scores. The degree of shading and the value reported correspond to the correlation between each pair of latent variables.

Pearson correlation coefficients were calculated to understand the relationships among latent variables (Figure 6). Negative correlations were observed between mirna1 and mirna2 (−0.47), REA and prot4 (−0.33), REA and prot2 (−0.3), carcass and prot4 (−0.31), carcass and prot2 (−0.28), and BFT and mirna3 (−0.25). Positive correlations are observed between all protein factors; meat quality and fat content (0.71), fat content and carcass (0.74), fat content and REA (0.76), and mirna2 and mirna3 (0.59). The latent variables REA and carcass correlated at 0.996. These results suggest that protein levels might have a negative impact on carcass, REA, and fat content factors.

### Bayesian network

**FIGURE 7:**
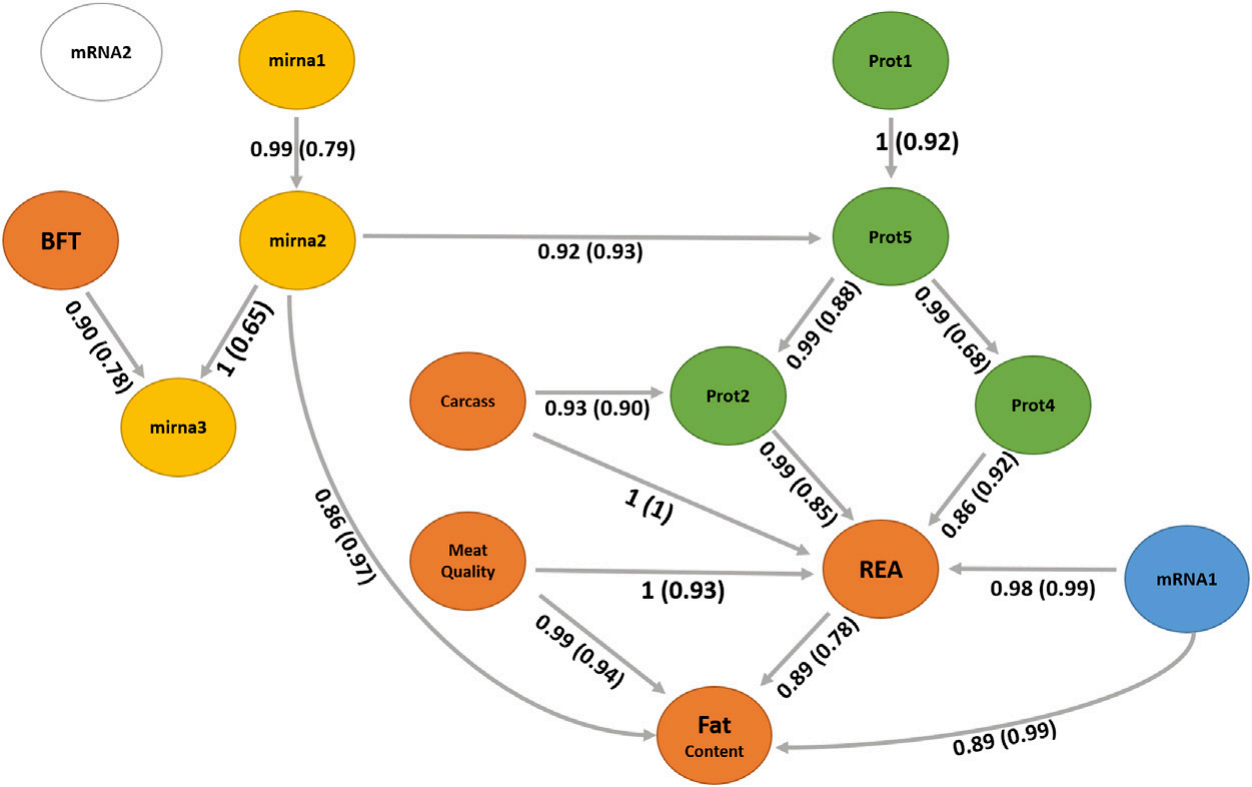
Bayesian network between latent variables based on the score-based (hill climbing and tabu) algorithms. The quality of the structure was evaluated by bootstrap resampling and model averaging across 1,000 replications. Orange nodes: phenotype latent variables; yellow nodes: miRNA latent variables; green nodes: protein latent variables; blue node: gene expression of mRNA1 module (WGCNA); and white node: gene expression of mRNA2 module (WGCNA). The labels of the arcs correspond to the strength and direction (in parenthesis).

A BN was used to infer the interrelationships between latent variables. The BN algorithm learned with the most favorable network score in terms of BIC (1801.31) and BGe (1903.46) was the score-based hill climbing algorithm (Figure 7). The structure of BN was refined by model averaging with 1,000 networks from bootstrap resampling to reduce the impact of local optimal structures. The labels of the arcs measure the percentage of the uncertainty, corresponding to strength and direction (in parenthesis). The strength measures the frequency of the arc presented among all 1,000 networks from the bootstrapping replicates and the direction is the frequency of the direction shown conditionally in the presence of the arc.

We observed no difference in the structures between the two score-based algorithms used, the hill climbing and tabu. The two score-based algorithms produced a greater number of edges than the hybrid algorithms. The hill climbing algorithm produced 17 directed connections from the 14 latent variables.

## Discussion

We integrated a multi-omic dataset with production, carcass, and meat quality traits and explored non-conventional relationships that led to new hypotheses in the meat quality field. Here, we applied EFA, BCFA, and BN to infer interrelationships among latent variables underlying complex traits and omic data. First, EFA and BCFA were used to reduce the dimensions of datasets by constructing latent variables and estimating their factor scores (de los Campos and Gianola, 2007). These latent variables represent more straightforward biological meanings than the original features measured in a population (Yu et al., 2019). Then, we applied a BN to understand the interrelationships among the latent variables (Neapolitan and others, 2004). We generated a network with 14 latent variables involving 17 directed connections. Moreover, this approach elucidated both direct and indirect relationships among latent variables. However, a precaution is essential to interpret the network as a causal relationship because causal statements require more assumptions (Pearl, 2009).

Yu et al. (2019) and Momen et al. (2021) applied a similar approach to obtain genetic insights on rice and wheat complex traits. Leal-Gutiérrez et al. (2018) studied the potential of using latent variables, obtained by structural equation analysis, on carcass and meat quality traits in beef cattle. They reduced the complexity of the data and reported biological mechanisms, such as postmortem proteolysis of structural proteins and cellular compartmentalization, cellular proliferation and differentiation of adipocytes, and fat deposition. In recent work, Yu, et al. (2020) applied factor analysis to beef cattle behavior to better understand latent factors underlying temperament traits.

### Biological meaning of latent variables and their relationships

The latent variable for the carcass, mainly composed of carcass cold weight, carcass depth, and fat kidney content (Supplementary Table S2), can be interpreted as the overall representation of the carcass, a higher value indicates a larger and heavier carcass. Its direct and indirect relationships with the latent variable REA (Figure 7) suggest the positive impact of the carcass yield on the ribeye area. Aass (1996) reported a positive phenotypic correlation (0.26) between the carcass depth and ribeye area corroborating our findings. Dinkel and Busch (1973) also estimated a positive genetic correlation (0.32) between growth rate and carcass yield, impacting the ribeye area positively.

The carcass has a relationship with the latent variable prot2 that also impacts REA. The latent variable prot2 is composed of 10 proteins (Supplementary Tables S2, S4), including UQCRC2 related with proteolysis (GO:0006508); ATP5F1A and ATP5F1B related with ATP synthesis coupled proton transport (GO:0042776 and GO:0015986); TNNT1 and TRIM72 related with muscle contraction (GO:0006936), regulation of muscle contraction (GO:0006937), sarcomere organization (GO:0045214), and muscle organ development (GO:0007517); GOT1 and GOT2 related with aspartate biosynthetic and catabolic processes (GO:0006532, GO:0006533), cellular response to insulin stimulus (GO:0032869), fatty acid homeostasis (GO:0055089), glutamate catabolic process to aspartate (GO:0019550), glycerol biosynthetic process (GO:0006114), and oxaloacetate metabolic process (GO:0006107); and MDH1 and MDH2 related with the carbohydrate metabolic process (GO:0005975), malate metabolic process (GO:0006108), NADH metabolic process (GO:0006734), oxaloacetate metabolic process (GO:0006107), tricarboxylic acid cycle (GO:0006099), and aerobic respiration (GO:0009060).

The ATP synthase F (0) complex subunit B1 (ATP5F1) has been positively correlated with meat color parameter a*, which impacts meat discoloration (Yu et al., 2017). Our findings show an indirect relationship between prot2 and the fat content latent variable that includes the parameter a*. Although prot2 and meat quality are not directly connected (Figure 7), both impact REA. However, prot2 is mainly composed of enzymes involved with energy metabolism that have been reported as putative candidate proteins for meat tenderness. The aspartate aminotransferase (GOT1) has been considered a putative candidate protein usable for meat tenderness prediction (Boudon et al., 2020). Rodrigues et al. (2017) reported that Nellore cattle have a higher abundance of malate dehydrogenase (MDH1) compared to Angus. This enzyme is important in gluconeogenesis, catalyzes the oxidation of malate to oxaloacetate, and is a relevant player in meat quality characteristics because this enzyme is involved in energy metabolism and affects how pH drops, changing the conversion of muscle to meat (Rodrigues et al., 2017). The ubiquinol-cytochrome C reductase core protein 2 (UQCRC2) gene, which is an important energy promoter for the development of cell functions was reported as up-regulated in a study analyzing gene expression on tough beef groups compared to the tender group in Nellore cattle (Muniz et al., 2021). The degradation of troponin T1 (TNNT1) proteins during post-mortem has been associated with meat tenderness (Zakrys-Waliwander et al., 2012; Contreras-Castillo et al., 2016; Wright et al., 2018).

The prot4 latent variable also shows a relationship with REA, which has two proteins (Supplementary Tables S2, S4) and includes the TNNI2 and MYH4. Troponin I, fast-twitch isoform (TNNI2) is a subunit of the troponin complex and plays a role in calcium regulation during muscle contraction and relaxation. The *TNNI2* gene was associated with pH, meat color value, and intramuscular fat content in pigs (Yang et al., 2010). The myosin heavy chains are relevant to muscle contraction velocity and power, MYH4 is one of the isoforms associated with IIb fibers types (Cho et al., 2016) and myotube hypertrophy in beef cattle (Bordbar et al., 2020). Our findings suggest new hypotheses of the impact of these proteins of prot2 could affect the REA and carcass traits.

The latent variable meat quality composed of shear force, myofibrillar fragmentation index, and the color parameters L* and b* can be interpreted as the overall representation of meat tenderness, and lower levels of this factor indicate more tender meat. It has a direct relationship with the latent variable REA and fat content. The relationship among tenderness, REA, and fat content has been discussed in the literature (Dinkel and Busch, 1973; Bonin et al., 2020). The mRNA1 latent variable has a relationship with REA and fat content. The mRNA1 factor is composed of the genes *LTN1, NFIA, ATP11B, FILIP1, RANBP2, N4BP2*, and *CERT1*. The nuclear factor IA gene (*NFIA*) has been studied indicating the potential to stimulate lipid accumulation in cattle (Chen H.-J. et al., 2018). According to the enrichment analysis (Supplementary Tables S5), these genes have been associated with an important cholesterol pathway called cholesterol and sphingolipid transport. Examples are the *RANBP2* gene which is associated with proteolysis and the *CERT1* gene which is related to intracellular cholesterol transport and sphingolipid metabolism. A further investigation is necessary to understand these relationships with REA or fat content.

The latent variable mirna3 is a child node of BFT and mirna2. The miRNAs are small RNA molecules that inhibit translation or induce degradation of protein-coding mRNAs that contain complementary sequences to miRNAs. mirna3 is constituted by three miRNAs, namely, bta. let.7g, bta. miR.26b, and bta. miR.423.5p. bta. let.7 g was found in studies related with lactation and infection in cattle (Ma et al., 2019; Rani et al., 2020). mirna2 is composed of two miRNAs, namely, bta. let.7e and bta. miR.339b. Gu et al. (2007) identified the expression of bta. let.7e on adipose tissue in cattle. bta. miR.339b was found in studies related to fatty acid metabolism and lactation (Do et al., 2017; Palombo et al., 2018; Poleti et al., 2018). mirna2 has a direct relationship with fat content (Figure 7). Further studies are necessary to better understand the functions of mirna2 and its association with fat metabolism in beef cattle.

The latent variable prot5 is composed of two isoforms of nebulin (NEBU) which are important structural components involved in meat aging (Koohmaraie et al., 1984; Ouali et al., 1995). Post-mortem degradation of nebulin has been associated with meat tenderness in cattle in which animals with a lower degradation have less tender meat (Anderson and Parrish, 1989; Wu et al., 2014). The prot5 latent variable is an important node that has relationships with prot2 and prot4 and has an indirect relationship with REA and fat content.

The generated network identified interomic relationships, bringing simplicity without losing complexity. This is one of the challenges found in studies of this nature. Often a methodology used ends up providing the interpretation of unfeasible results, which was not in our approach. Additional investigations are essential to understand the relationships of molecules and phenotypes on latent variables REA, prot2, prot4, prot5, mRNA1, carcass, mirna3, mirna2, and fat content. The network demonstrated a relationship between miRNAs and nebulin protein isoforms that will not be found in studies using single or multi-level omics. Finally, REA appears as a central node in the network, influenced by carcass, prot2, prot4, and meat quality, suggesting that REA is a good indicator phenotype for meat quality because it can be easily measured during slaughter or by ultrasonography.

## Conclusion

The FA identified latent variables, decreasing the dimensionality and complexity of data. The BN analysis was capable of identifying interrelationships among latent variables from different types of data, allowing the integration of different types of omic data and complex traits. The EFA, BCFA, and BN approaches can be used to generate new hypotheses on molecular research in the meat quality area, by integrating different types of data and exploring non-conventional relations.

## Data Availability

The mRNA datasets supporting the conclusion of this article are available in the European Nucleotide Archive (ENA) repository (EMBL-EBI), under accession nos. PRJEB13188, PRJEB10898, PRJEB15314, and PRJEB19421 (https://www.ebi.ac.uk/ena/browser/view/). The miRNA dataset of this article is available in the European Nucleotide Archive (ENA) repository (EMBL-EBI), under accession no. PRJEB42280. The protein data presented in the study are publicly available. These data can be found at: https://doi.org/10.1016/j.dib.2018.06.004. Any other relevant data are available from the authors upon reasonable request.
